# Fermentation with Edible *Rhizopus* Strains to Enhance the Bioactive Potential of Hull-Less Pumpkin Oil Cake

**DOI:** 10.3390/molecules25245782

**Published:** 2020-12-08

**Authors:** Anna Starzyńska-Janiszewska, Robert Duliński, Bożena Stodolak

**Affiliations:** Department of Biotechnology and General Technology of Food, Faculty of Food Technology, University of Agriculture in Krakow, Balicka 122, 30-149 Krakow, Poland; robert.dulinski@urk.edu.pl (R.D.); bozena.stodolak@urk.edu.pl (B.S.)

**Keywords:** antioxidant potential, inositol phosphates, tempe-type fermentation, hull-less pumpkin oil cake, agro-industrial by-products

## Abstract

Solid-state fermentation with food-grade fungal strains can be applied to enhance the bioactive parameters of agro-industrial by-products. Tempe-type fermentation can be adapted to various substrates, but the key factor is the appropriate strain selection. The aim of this study was to compare the potential of *Rhizopus* strains for obtaining products of improved antioxidant activity from pumpkin oil cake. For this purpose, substances reacting with the Folin-Ciocalteu reagent, with free radical scavenging potential, as well as reducing power were assessed. The effect of the fermentation on the phytate level and inositol phosphate profile in the material was also monitored. The fermentation resulted in the significant enhancement of the antioxidant potential of pumpkin oil cake in the case of all the strains tested, but the most efficient one was *R. oligosporus* ATCC 64063. During the course of fermentation, the level of phytate in the material decreased (the highest reduction rate was observed in the oil cake fermented with *R. oryzae* CBS 372.63), while peptides and fungal glucosamine were accumulated. Tempe-type fermentation can be considered as an alternative way of improving the bioactive parameters of pumpkin oil cake and, thanks to the various activities of different *Rhizopus* strains, it is possible to obtain products of desired parameters.

## 1. Introduction

Solid-state fermentation with fungal strains has been traditionally used in order to obtain various plant foods of advantageous nutritional and bioactive parameters. Nowadays, it is also considered an alternative efficient and economically viable method of enhancing the bioactive content of agro-industrial waste [1]. An interesting example of solid-state fermentation of plant materials is tempe-type procedure with the use of *Rhizopus* sp., mainly *R. oligosporus* and *R. oryzae*, which has its origin in the Indonesian region. The main substrate is legume seeds (soy), alone or mixed with cereal grains. A similar procedure is also applied in West Java to process by-products of tofu production (okara), as well as coconut and peanut oil production, into food products called ‘black oncom’ and ‘red oncom’, after fermentation with *Rhizopus* sp. or *Neurospora* sp., respectively. The result of the fermentation is partial digestion of substrate macromolecules (protein, carbohydrates, lipids), decomposition of antinutrients and increased availability of bioactive compounds, as well as their biotransformation and synthesis [2].

Tempe-type fermentation is a relatively simple technique that can be adapted for different plant materials intended for new food products and valuable food additives. Our previous research proved that it can be successfully applied to obtain products of high nutritional value and antioxidant activity from non-conventional raw materials (pseudo-cereals, grass pea seeds) and by-products (flaxseed oil cake) with the use of *R. oligosporus*, *Aspergillus oryzae*, and *Neurospora intermedia* strains [3,4,5]. The results described in the papers cited showed that an appropriate fungal strain selection is in this case the key strategy to take full advantage of the potential of a given substrate.

Pumpkin oil cake is a waste product of oil cold-pressing from the seeds of hull-less (naked) pumpkin (*Cucurbita pepo*). The seeds have only a thin membranous seed coat, which allows the extraction of a higher yield of oil. Apart from being mainly used as animal feed and fertiliser, pumpkin oil cake is edible and has qualities which make it valuable in food production [6]. It is characterised by a high nutritional value (around 60%–70% of easily-digestible protein rich in essential amino acids) and contains more phenolic acids of better extractability than pumpkin seeds [7]. The level of antinutrients in pumpkin oil cake is generally low, as measured by Zdunczyk et al. [8]: 1330 TUI/g trypsin inhibitor activity and 19.9 mg/g α-galactosides, with the exception of inositol phosphates—40.5 mg/g, which is over three times more than in soybean meal. Inositol phosphates, despite of their negative role in digestion (at the physiological pH, phytates form insoluble salts and complexes with minerals and macronutrients, thus limiting their absorption) are nowadays usually perceived as desirable functional food components. According to recent studies, InsP6 and lower forms of inositol phosphates (InsP1–5) may have important physiological functions. They showed anti-inflammatory and anticancer activity in various types of cell lines, as well as antioxidant and antidiabetic properties. Supplementation with inositol and IP6 may help reduce free radical cellular damage, e.g., lipid peroxidation, since it reverses the diabetes-induced downregulation of the antioxidant defence mechanisms [9].

Examples of the usage of pumpkin-oil cake in the food industry are rather rare. Defatted pumpkin flour has been used as an ingredient of cookies (as a substitute of up to 10% of wheat flour) and thus considered a potential nutritional supplement [10]. Pumpkin oil cake has also been recognised as a promising source of protein hydrolysates of antioxidant and functional properties [6] and biodegradable edible films [11]. At the same time, this by-product can be taken into consideration as a substrate for tempe-type fermentation that may potentially improve the bioactive content of the tempe product. Due to the high level of protein and, on average, 12% fat content [8], pumpkin oil cake is expected to be a good substrate for the growth of *Rhizopus* strains. Pumpkin seed flour was previously proven to be a promising substrate (without adding nutrients) for lipase production with the use of *Aspergillus niger* [12].

The purpose of the present study was to compare the potential of food-grade *R. oligosporus* and *R. oryzae* strains for obtaining products of improved antioxidant activity from pumpkin oil cake. The effect of the fermentation on the inositol phosphate profile in the material, as well as other fermentation parameters (protein, peptides, glucosamine), was also monitored.

## 2. Results and Discussion

### 2.1. Antioxidant Activity

Because of the complex structure of fermented food materials, their antioxidant potential is generally not due to one specific molecule but the net effect of the interaction of different compounds, such as phenols, reducing sugars, peptides, and amino acids. Even within one group of compounds, such as flavonoids, antagonist and synergic effects were observed for food extracts [13]. Thus, the application of several analytical methods allowing for the estimation of different aspects of the antioxidant activity is in this case an appropriate approach to assess the overall potential of the products tested. The main emphasis was placed on studying the activity of aqueous (buffer) extracts because they can be considered analogous to physiological conditions. The use of extracts prevented the loss of the active compounds that could have occurred during the evaporation of the solvent due to oxidation and/or denaturation. In buffer extracts, ABTS˙^+^- and ˙OH- scavenging assays, as well as the level of Folin-Ciocalteu reacting substances (FCRS) and reducing power, were determined.

The raw pumpkin oil cake contained 3.33 mg/g DM FCRS (Table 1), which was more than 2.61 mg/g DM reported in pumpkin meal by Zdunczyk et al. [8]. The assay with the Folin-Ciocalteu reagent is usually applied to determine the total level of polyphenols in the material, but the extracts from pumpkin oil cake could have contained different water-soluble molecules capable of reducing the Folin-Ciocalteu reagent. According to Peričin et al. [7], hull-less pumpkin oil cake obtained from a Serbian producer had 0.043 mg free phenolic acids and 0.039 mg esterified phenolic acids in g DM.

#### 2.1.1. Effect of the Processing

The level of FCRS measured in the water extracts was significantly correlated with the activity against ABTS˙^+^ (R^2^ = 0.947) (Table 1). ABTS neutralization assay is a basic antiradical method popularly used to measure the antioxidant potential of dietary antioxidants and foods [14]. Both autoclaving and the subsequent fermentation of pumpkin oil cake with *Rhizopus* strains resulted in a significant increase in the antiradical activity and FCRS. The products obtained after the 48-h fermentation had the ABTS˙^+^-scavenging capacity and the FCRS level increased, on average, by 124% and 214%, respectively, as compared to the raw material. In most cases, however, the prolongation of fermentation (up to 96 h) did not cause any further significant improvement. As can be seen from the comparison of the results of the ABTS˙^+^-scavenging method measured in buffer extracts (SA-ABTS˙^+^) and the ones obtained from dried material directly introduced into the radical solution (Quencher-ABTS˙^+^, QA-ABTS˙^+^), almost the whole pull of compounds present in the fermented products was water-extractable (Table 1). Slightly lower values were only noted for pumpkin oil cake fermented with *R. oryzae* (up to 80%). In the case of raw and autoclaved pumpkin oil cake, the antioxidant potential available in buffer extractions accounted for 41% and 73% of the QA-ABTS˙^+^ results, respectively. Thus, tempe-type processing was proven to be effective in converting antioxidants present in the raw pumpkin oil cake into their easily mobilised forms. During fermentation, microbial enzymes release molecules bound within the plant matrix, making them more available for the extraction and increasing their activity (e.g., the number of free hydroxyl groups in the phenyl ring of phenolic aglycones). As shown by Ratz-Łyko and Arct [15], after the hydrolysis of pumpkin oil cake with microbial β-glucanase and β-glucosidase, the level of FCRS and reducing sugars increased in the extracts, which was accompanied by a significant rise in the antiradical activity. The proteolytic activity of the fungal strain during the fermentation of plant substrates can also result in the production of low-molecular weight peptides with antioxidant activity [16].

The hydroxyl radical scavenging assay used in this study is preferentially suited for water/buffer extracts [17]. The hydrogen radical scavenging activity (SA-˙OH) resulted from the direct neutralization of ˙OH and/or the ability to chelate Fe ions necessary to generate hydroxyl radicals in the reaction medium. ˙OH is the most active free radical, which can react indiscriminately with any oxidizable molecule present in the environment, including lipids, peptides, nucleotides, sugars, and phenols. Within the polyphenol group, molecules with the available *ortho-* position in the phenolic ring are considered effective hydroxyl radical scavengers [18]. Autoclaving greatly improved the SA-˙OH in pumpkin oil cake, as the measured EC_50_ was 3,6-fold lower (0.42 mg DM) than that of the raw substrate (Table 1). In contrast, the subsequent fermentation had a negative effect on this parameter. After 48 h of incubation, EC_50_ values of the material increased within the range of 21% (*R. oligosporus* ATCC 64063) to 118% (*R. oryzae*) and this tendency generally continued through the course of fermentation (the exception was only the oil cake subjected to 96-h fermentation with the *R. oryzae* strain). Nevertheless, it should be stressed that all the fermented products have a significantly higher power against ˙OH than the raw pumpkin oil cake (EC_50_ 0.51–1.13 vs. 1.52). The effect of tempe-type fermentation on the ˙OH scavenging ability of plant materials (flaxseed oil cake, quinoa seeds, buckwheat groats) observed in our earlier experiments was positive [19,20,21]. However, the prolongation of the incubation (up to 144 h) was not effective in the case of flaxseed oil cake fermentation with *R. oligosporus* DSM 1964 and ATCC 64063 [19]. A result similar to the one observed in the present study was previously reported for sugared black tea fermented with kombucha (‘tea fungus’—a symbiotic association of acetic acid bacteria and yeasts), where the ˙OH-scavenging activity decreased, while the DPPH˙-scavenging activity increased as a result of biotreatment [22].

Fermentation with the *Rhizopus* strains was effective in improving the reducing power of the material (Table 1). RP_0.5_ measured for fermented pumpkin oil cake was 2.5–4.5-fold lower than that of the autoclaved sample (and 2.32–4.11-fold lower than in raw oil cake, as thermal treatment decreased the reducing power of the substrate). The prolongation of the incubation time (up to 72 and 96 h) did not further improve the activity of the material, and it even had the opposite effect in the case of two strains (RP_0.5_ values increased by over 30% (DSM 1964) and over 70% (ATCC 64063), as compared to the material fermented for 48 h). This tendency is different than the one observed during the fermentation of flaxseed oil cake with ATCC 64063 and DSM 1964, where the longer incubation time (72 h–144 h) was generally more effective than a two-day process [19].

#### 2.1.2. The Comparison of the Effect of Individual Strains

All the tested *Rhizopus* strains proved to effectively improve the antioxidant potential of pumpkin oil cake during the 48-h fermentation, which was the shortest incubation time necessary for the fungal strains to overgrow the material. The best results were obtained with *R. oligosporus* ATCC 64063 (Table 1). The pumpkin oil cake fermented with this strain for two days exhibited activity against ABTS˙^+^ and ˙OH higher by, on average, 90% and 54%, respectively, as well as 21% more FCRS than the products obtained with the use of the other fungi. As to the reducing power, the 48-h fermentation with ATCC 64063 was also the most effective among all the variants tested, resulting in around 30% lower RP_0.5_ of the product. This strain had been initially recommended for the production of cereal-based tempe due to its scarce amylolytic activity, which ensures that tempe products do not contain undesirable organic acids derived from glucose metabolism [23]. It can be seen, however, that *R. oligosporus* ATCC 1964 may be considered a versatile strain also suitable for the fermentation of pseudo-cereals, such as quinoa seeds, buckwheat groats [4,21], as well as edible waste from cold-pressed oil extraction, such as pumpkin oil cake (the present study) and flaxseed oil cake [19]. With regard to the ability to increase both the level of soluble phenols and the antioxidant activity of the said substrates, it showed a better potential than other *R. oligosporus* strains tested here (DSM and especially NRRL), as well as strains that belong to different edible filamentous fungi species used in traditional Asian food fermentations: *Rhizopus oryzae* (the present study), *Aspergillus oryzae*—koji strain, and *Neurospora intermedia*—oncom strain [4]. It can also be mentioned here that *R. oryzae* CBS 372.64 which was the least efficient one among all the tested strains, was also originally isolated from tempe. Different strains belonging to *R. oryzae* were previously reported to have good potential for improving the polyphenolic content and antioxidant properties of cereals and agro-industrial residues (bran rice) [24].

### 2.2. Inositol Phosphates

The level of phytate and the inositol phosphate profile are presented for the selected variants as our focus was mainly on the fungal activity and the difference between the shortest (48 h) and the longest (96 h) fermentation time.

#### 2.2.1. Phytate

**Figure 1:**
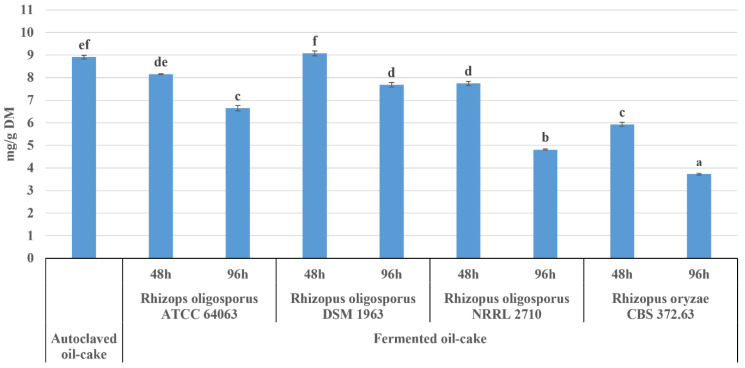
The level of phytate in autoclaved and fermented pumpkin oil-cake. One-way analysis of variance and Tuckey post-hoc test were applied. Data is shown as the mean ± SEM. Different letters depict data that differ significantly (*p* ≤ 0.05).

The level of phytate measured in autoclaved pumpkin oil cake using HPAEC chromatography coupled with UV/VIS detection was 8.91 mg/g DM (Figure 1). The obtained result is comparable with data reported by Simonet et al. [25] for raw pumpkin seeds (9.43 mg/g), determined by flow injection-capillary zone electrophoresis. These amounts are much smaller than 23.7 mg/g and 30.1 mg/g measured in defatted pumpkin flours by El-Adawy and Taha [26] and Mansour et al. [27], respectively.

The solid-state fermentation of autoclaved pumpkin oil cake caused a statistically significant decrease in the phytate content, with the exception of material fermented for 48 h with *R. oligosporus* DSM 1964. The most effective strain was *R. oryzae*, whose activity resulted in a 34% decrease after two days of fermentation, and by another 24% after the next 48 h (3.79 mg/g). A comparable dynamics of inositol polyphosphate degradation was shown by *R. oligosporus* NRRL 2710, although in this case the total reduction after 96 h reached the level of 47%. The least efficient were *R. oligosporus* DSM 1964 and ATCC 64063, for which a decrease by 14% and 24%, respectively, was observed after 96 h of incubation. Interestingly, these data differ from the results of our previous experiment, where the ATCC strain was proven more effective than DSM 1964 during the fermentation of flaxseed oil cake [28]. This supports the observations of Ramachandran et al. [29] and Rani et al. [30] according to which the efficiency of phytase production by *R. oligosporus* and *R. oryzae* strains differs significantly depending on the type of oil cake. However, based on the results of the present study and data shown by Ramachandran et al. [29], *R. oryzae* strains can be regarded as a promising tool for decreasing the phytate level in this type of agro-industrial by-products.

#### 2.2.2. Inositol Phosphate Profile

**Figure 2:**
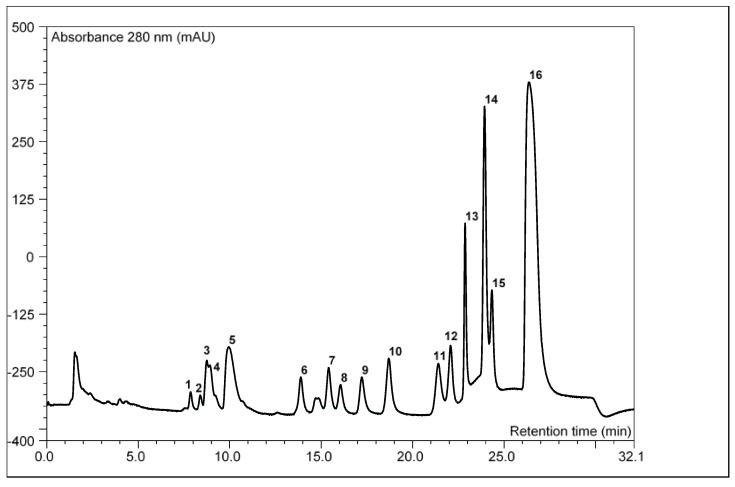
Exemplary high-performance anion-exchange chromatography (HPAEC) chromatogram of inositol phosphate profile of fermented pumpkin oil-cake (48-h fermentation with *Rhizopus oligosporus* ATCC 64063). 1—Ins(2,4,6)P3; 2—InsP3; 3—InsP3; 4—Ins(2,4,5);(1,4,5)P3; 5—InsP3; 6—InsP4; 7—InsP4; 8—InsP4; 9—InsP4; 10—InsP4; 11—Ins(2,4,5,6)P4; 12—Ins(1,2,3,4,6)P5; 13—Ins(1,2,3,4,5)P5; 14—InsP5; 15—DL-Ins(1,2,4,5,6)P5; 16—phytate.

The profile of the inositol phosphates in the autoclaved pumpkin oil cake showed a dominant share of inositol hexaphosphate (69%) and inositol pentaphosphate (InsP5) (19%) (Table 2, Figure 2). The share of InsP6 in the profile can be even higher, as presented by Simonet et al. [25] for raw pumpkin seeds (80% InsP6 and 12% InsP5). The first 48 h of fermentation caused changes in the distribution of lower phosphate profiles and a reduction in the share of InsP6. The most significant decrease in InsP6 (approx. 50%) was recorded for the *R. oryzae* strain, with an increased share of compounds with phosphate groups 3 (18%) and 4 (40%), e.g., in the configuration Ins(2,4,5);(1,4,5) P3 and Ins (2,4,5,6) P4, the former of which may play an important role in nerve transmission [31]. After 96-h incubation, the increase in the percentage of phytate in the entire spectrum at the expense of lower inositol phosphates was observed (the exception was the material fermented with *R. oryzae*). The described phenomenon could have resulted from the reduction of the metabolic activity of mycelium and the conversion of mainly lower InsP1–4 phosphates in a later stage of fermentation. The intensity of phytase synthesis by fungal strains can change throughout the incubation period under the conditions of solid-state fermentation. As demonstrated by Ramachandran et al. [29], the phytase production by the *R. oryzae* strain in mixed substrate (coconut and sesame oil cakes) gradually increased with time until the 72nd hour of incubation at 30 °C, and then decreased.

### 2.3. Protein, Peptide and Glucosamine Levels

**Figure 3:**
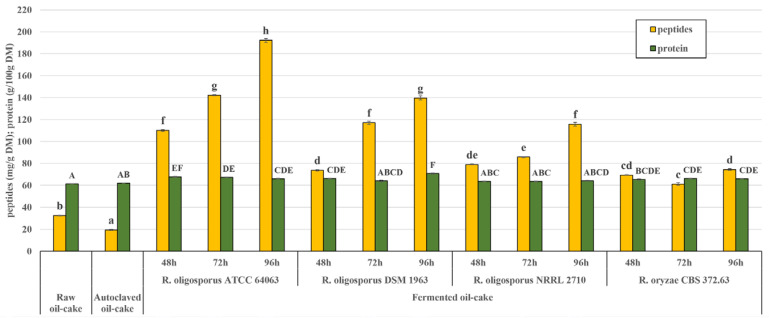
Protein and peptides in pumpkin oil-cake—raw, autoclaved and fermented with *Rhizopus* strains. One-way analysis of variance and Tuckey post-hoc test were applied. Data is shown as the mean ± SEM. Different letters depict data that differ significantly (*p* ≤ 0.05) within the parameter.

**Figure 4:**
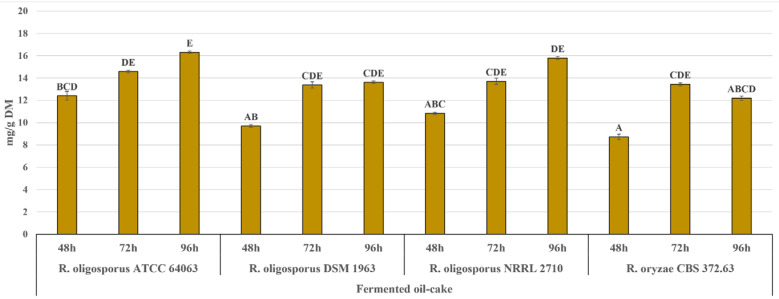
Glucosamine in pumpkin oil-cake fermented with *Rhizopus* strains. One-way analysis of variance and Tuckey post-hoc test were applied. Data is shown as the mean ± SEM. Different letters depict data that differ significantly (*p* ≤ 0.05).

When considering the application of the tempe-type fermentation to improve the bioactive potential of pumpkin oil cake, it seems advisable to also examine the level of protein and protein hydrolysis products in the material, as this substrate is originally a good protein source. The raw material used as a substrate in the present experiment contained 61% of crude protein (Figure 3). Solid-state fermentation can result in the enrichment of agro-industrial by-products in proteins, as shown for rapeseed meal fermented with the *R. oligosporus* strain (an increase of up to 34%) [32]. In the present experiment, the level of crude protein after the tempe-type fermentation increased slightly, by 6% on average. The fermentation also raised the level of peptides in the material, which increased gradually during 96 h of incubation (Figure 3). The release of peptides as a result of fermentation indicates the proteolytic activity of the fungal strain. Moreover, increasing the peptide content could enhance the antioxidant potential of the material. Based on the recent research, oilseed meals contain bioactive peptides encrypted within their protein sequence, which can be released by microbial enzymes and applied as natural food ingredients [33]. Fungal (*Aspergillus niger*) protease was shown to improve the antiradical properties of hull-less pumpkin oil cake protein isolate [34]. In the presented experiment, the content of peptides in the material was significantly correlated with the level of FCRS (R^2^ = 0.897) and ABTS˙^+^-scavenging activity (R^2^ = 0.857 for SA-ABTS˙^+^ and 0.844 for QA-ABTS˙^+^). The most dynamic accumulation of peptides was observed in the case of the ATCC 64063 strain. The level of peptides in the material corresponded with the content of fungal glucosamine (Figure 4), which also increased through the course of the incubation (R^2^ = 0.616). Higher amounts of glucosamine were measured in pumpkin oil cake fermented with *R. oligosporus* ATCC 64063 and NRRL 2710 (up to 15 mg/g) than in the case of *R. oligosporus* DSM 1967 and *R. oryzae* CBS 372.63 (up to 12.4 mg/g). Glucosamine is a good indicator of fungal biomass growth under the conditions of solid-state fermentation [35]. Moreover, it is worth mentioning that the *Rhizopus* cell wall composition belongs to the chitin-chitosan category, and contains up to three times more chitosan than chitin [36]. These both components are considered valuable sources of dietary fibre and functional ingredients. Chitosan in particular is proven to exert hypocholesterolemic and lipid-lowering activity and in some countries, it is treated as a food quality enhancer. In Japan, for example, dietary cookies, potato chips and noodles enriched with chitosan are available on the market [37].

## 3. Materials and Methods

### 3.1. Materials

The substrate used in the fermentation process was cold-pressed oil cake from hull-less pumpkin seeds kindly provided by “PPHU Maszyny i Przetwórstwo Nasion Oleistych Ol’Vita” (Pszenno, Poland). The material was stored at 4 °C and used for the fermentation within one month.

### 3.2. Microorganisms

Tempe strains of *Rhizopus oligosporus* ATCC 64063, DSM 1964 and NRRL 2710, as well as *Rhizopus oryzae* CBS 372.63 were grown on potato extract agar (PDA) for 12 days at 24 °C. Then, the spore suspension of each strain was obtained in sterile saline solution (8 g/L, supplemented with peptone (0.01 g/L) and Tween 80 (0.1 mL/L)) and filtered three times through nylon net filters (ϕ 11 µm, Millipore, Cork, Ireland) in order to remove fragments of mycelium. The spore density was obtained by spore-counting in a Thoma Chamber.

### 3.3. Fermentation of Pumpkin Oil Cake

Ground (pore diameter in the grinder 0.5 mm) pumpkin oil cake was autoclaved (121 °C, 20 min). After cooling to room temperature, the material was hydrated to 60% moisture content with sterile distilled water and simultaneously acidified to pH 4–5 with an appropriate quantity of 5% (*w*/*v*) lactic acid. Then, the substrate was thoroughly mixed with the spore suspension of *R. oligosporus* ATCC 64063, *R. oligosporus* DSM 1964, *R. oligosporus* NRRL 2710 or *R. oryzae* CBS 372.63 (3 × 10^6^ spores per 100 g of raw substrate). Inoculated material was tightly packed in Petri dishes (ø 11 cm, four Petri dishes for each fermentation period) and incubated at 37 °C (the first 2 h—induction of spore germination), followed by 30 °C in 50% air humidity. Fermented products were steamed (10 min), lyophilized and stored at −20 °C until analysed.

The analyses were conducted on the following stages of pumpkin oil cake processing: raw, autoclaved, and fermented with *Rhizopus* strains for 48, 72, and 96 h.

### 3.4. Analytical Methods

Extract Preparation. Lyophilized material was used to prepare extracts at a concentration of 0.5 g/25 mL in sodium-phosphate buffer (0.02 mol/L, pH 7.4) by 2-h-long shaking, centrifugation, and filtration of the supernatant.

Antioxidant methods. Folin-Ciocalteu Reacting Substances (FCRS) (mg gallic acid/g DM) were determined in buffer extracts by the method of Swain and Hillis [38]. The ABTS˙^+^ scavenging activity (SA-ABTS˙^+^) was measured in buffer extracts according to Arnao et al. [39]. The ABTS˙^+^ solution (10 mg ABTS [2,20-azino-bis(3-ethylbenzothiazoline-6-sulfonic acid)] dissolved in 1.3 mL 0.0049 mol/L K_2_S_2_O_8_ and 1.3 mL distilled water) was diluted with phosphate buffer to achieve the absorbance of 0.7 ± 0.02 at 734 nm. 2 mL of the ABTS˙^+^ solution was mixed with 200 μL extract and incubated at room temperature in the dark. After 7 min, the absorbance was measured at 734 nm, with phosphate buffer as a reference. The ABTS˙^+^ scavenging activity was expressed as mg Trolox equivalents/g DM.

The quencher-ABTS˙^+^ assay (QA-ABTS˙^+^) was performed according to Gökmen et al. [40]. The lyophilized material (10 mg) was mixed with 30 mL of the ABTS˙^+^ solution (initial absorbance 0.7 ± 0.02 at 734 nm), and shaken at room temperature in the dark. After 7 min, the absorbance of filtered samples was measured at 734 nm, with phosphate buffer as a reference. The results were expressed as mg Trolox equivalents/g DM. The hydroxyl radical neutralization (SA-˙OH) was obtained in buffer extracts according to Marambe et al. [41]. Briefly, 50–600 μL extract was mixed with an appropriate volume of potassium phosphate buffer (20 mmol/L, pH 7.4) to achieve 1125 μL. Next, the following components were added: 40 μL 0.5 mmol/L FeCl_3_, 42 μL 2.4 mmol/L EDTA, 140 μL 0.02 mol/L deoxyribose, 10 μL 0.01 mol/L ascorbic acid and 142 μL 1 mmol/L H_2_O_2_. The assay mixture was incubated at 37 °C for 1 h, and after that 1 mL TBA (1%, *w*/*v*) and 1 mL TCA (2.8%, *w*/*v*) were added. The sample was incubated at 100 °C for 20 min, and after cooling to room temperature, the absorbance was measured at 532 nm against a blank. The blank consisted of all the reagents, to which TBA and TCA were added prior to incubation at 37 °C. The SA-˙OH was expressed as EC_50_ (efficient concentration), which refers to mg DM of the sample used for the extraction that is required for the inhibition of 50% of the free radicals under reaction conditions. A lower value of EC_50_ indicates higher radical scavenging activity. The reducing power was determined in buffer extracts according to Ardestani and Yazdanparast [42]. Briefly, 200–1500 μL extract was mixed with an appropriate volume of sodium phosphate buffer (0.2 M, pH 6.6) to achieve 3500 μL. Next, 2500 μL potassium ferricyanide (1%, *w*/*v*) was added and the mixture was incubated at 50 °C for 20 min. Then, 2500 μL TCA (10%, *w/v*) was added, and after centrifugation (3 kg, 10 min), 2500 μL of the supernatant was combined with 2500 μL distilled water and 500 μL ferric chloride (0.1%, *w/v*). After a 10-min incubation at room temperature, the absorbance of the solution was measured at 700 nm. The reducing power was expressed as RP_0.5_, defined as the amount of a lyophilized sample (mg DM) used for the extraction that produces 0.5 absorbance units at 700 nm. A lower value of RP_0.5_ indicates a higher reducing power.

The extraction of inositol phosphates from samples was conducted according to Duliński et al. [43]. The inositol phosphate profile was determined by the analytical system using high-performance anion-exchange chromatography (HPAEC) with postcolumn derivatisation (ISO-3000 Pump, Dionex, Sunnyvale, CA, USA) and UV/Vis detection (PDA-3000 Detector, Dionex, Sunnyvale, CA, USA) [44]. A reference sample was prepared by dissolving 2.3 g of sodium phytate in deionised water (50 mL) and adjusting the pH to 4.0 with 2 mol/L HCl. The solution was autoclaved for 40 min at 121 °C under 101 kPa (autoclave ELMI ESS-207; SMS Sp. z o.o.). The elution sequence of InsP6-2 isomers was established according to the work of Blaabjerg et al. [44], using an appropriate standard solution, sodium phytate (InsP6), inositol-(1,2,4,5,6)-pentakisphosphate, Ins(1,2,4,5,6)P5, inositol-(1,4,5,6)-tetrakisphosphate, Ins(1,4,5,6)P4, inositol-(1,3,4,5)-tetrakisphosphate, Ins(1,3,4,5)P4, inositol-(1,4,5)-trisphosphate, Ins(1,4,5)P3, inositol-(1,3,4)-trisphosphate, Ins(1,3,4)P3, and myo-inositol 2-monophosphate (all purchased from Sigma-Aldrich, St. Louis, MO, USA).

Phytate (mg/g DM) was estimated by the ion chromatography system (Dionex UltiMate 3000) coupled with an ED50a electrochemical detector and a conductivity cell (Dionex, Sunnyvale, CA, USA). Samples extracted according to Duliński et al. [43] were separated on Omnipac Pax-100 anion exchange column (250 mm × 4 mm i.d.) connected in series with Omnipac Pax-100 (8 mm × 1 mm) guard column (Dionex, Sunnyvale, CA, USA). A mobile phase consisting of a mixture of 200 mmol/L sodium hydroxide (A), deionised water (B), and water-isopropanol (50:50, *v/v*) (C) was used. An anion micromembrane suppressor AMMS 300 4-mm (Dionex, Sunnyvale, CA, USA) system was used to suppress the mobile phase conductivity before entering the conductivity cell (regenerant 0.25 mol/L sulfuric acid) according to Dionex Application Note 65 [45].

Crude protein (g/100 g DM) was determined on the basis of nitrogen level according to the Nessler method [46] in previously mineralized samples (Hach Digesdahl Digestion Apparatus at 280 °C (Hach Company, Loveland, CO, USA)). The nitrogen level was multiplied by 5.5. Total peptides (g/100 g DM) were measured in buffer extracts according to the o-phthaldialdehyde method as described in Zhu et al. [47], with a standard curve prepared from L-glutathione (reduced form). Briefly, 50 µL of a buffer extract was added to 2500 µL of the OPA mixture (the mixture composition: 20 mL 100 mM sodium tetraborate, 2.5 mL sodium dodecyl sulfate (20%, *w/w*), 40 mg o-phthalaldehyde dissolved in 1 mL methanol, 100 µL mercaptoethanol, and 21.4 mL distilled water). After a 20-min incubation at room temperature the absorbance at 340 nm was measured. The glucosamine level (g/100 g DM) in previously hydrolysed samples [48] was assessed using the colorimetric method described by Tsuji et al. [49]. 1000 µL of a hydrolysed sample was mixed with 1000 µL potassium bisulfate (5%, *w/v*) and 1000 µL sodium nitrite (5%, *w/v*), and incubated at room temperature for 15 min. Next, 1000 µL of ammonium sulfamate (12.5%, *w/v*) was added, followed by a 5-min incubation with shaking (800 rpm). Then, 1000 µL of MBTH solution (0.5% (*w/v*) 3-methyl-2-benzothaizolinone hydrazone hydrochloride hydrate) was added and the mixture was incubated at 100 °C for 4 min, and after that 1000 µL FeCl_3_ (0.5%, *w/v*) was added. The absorbance was read after a 40-min incubation at room temperature. A standard curve was prepared from glucosamine obtained after hydrolysis of N-acetyl-D-glucosamine [48]. To calculate the fungal glucosamine, the background level of seed glucosamine was subtracted from the total glucosamine content in the material. Dry matter (DM) was obtained with a moisture analyser (WPS 110S, Radwag, Radom, Poland).

### 3.5. Statistical Analysis

Experimental data were subjected to one-way analysis of variance (ANOVA) and expressed as a mean ± standard error of the sample (SEM). The Tukey post-hoc test was applied (*p* ≤ 0.05) to determine statistically significant differences. Data were processed using Statistica for Windows, ver. 13.1 (Statsoft, Tulsa, OK, USA) statistical software.

## 4. Conclusions

Solid-state tempe-type fermentation with *Rhizopus oligosporus* (ATCC 64063, DSM 1967, NRRL 2710) and *Rhizopus oryzae* (CBS 372.63) efficiently improved the bioactive potential of the hull-less pumpkin oil cake. A relatively short incubation time (48 h) was sufficient to obtain products of significantly higher antioxidant parameters, as compared to the raw substrate. Among the fungal strains tested, *R. oligosporus* ATCC 64063 was considered the most effective one. Pumpkin oil cake fermented with this strain for two days was characterised by ABTS˙^+^-scavenging activity higher by 368% (91% of which was easily extracted into water from plant matrix), 293% more FCRS, as well as higher ˙OH-scavenging activity (3-fold lower EC_50_) and reducing power (4-fold lower RP_0.5_) than the raw substrate. The material fermented with the ATCC strain also contained the highest amount of peptides, which were accumulated throughout the course of fermentation together with fungal glucosamine. The fermentation resulted in a slight enrichment of the product in crude protein.

Fungal activity also caused a gradual decomposition of inositol phosphates in pumpkin oil cake. In this case, the greatest changes were obtained with the use of the *R. oryzae* strain, resulting in a 34% (48-h fermentation) to 58% (96-h fermentation) reduction, as compared to the non-fermented material.

## Figures and Tables

**Table 1:** Antioxidant activity of pumpkin oil-cake—raw, autoclaved and fermented with *Rhizopus* strains.

			FCRS (mg/g DM)	SA-ABTS˙^+^ (µmol Trolox/g DM)	QA-ABTS˙^+^ (µmol Trolox/g DM)	SA-OH˙ (EC_50_)	Reducing Power (RP_0.5_)
Raw oil-cake			3.33 ± 0.09 a	18.25 ± 0.42 a	44.48 ± 0.77 a	1.52 ± 0.02 g	23.62 ± 0.51 f
Autoclaved oil-cake			4.79 ± 0.08 b	32.07 ± 0.22 b	43.94 ± 1.22 a	0.42 ± 0.02 a	25.72 ± 0.60 g
Fermented oil-cake	Strain	Time					
	*Rhizopus oligosporus* ATCC 64063	48 h	9.74 ± 0.05 f	67.16 ± 0.67 i	61.27 ± 1.20 de	0.51 ± 0.06 ab	5.74 ± 0.11 a
		72 h	10.14 ± 0.16 f	66.19 ± 0.80 i	64.61 ± 1.13 e	0.77 ± 0.05 cd	10.41 ± 0.16 e
		96 h	11.22 ± 0.13 g	68.34 ± 2.55 i	64.80 ± 0.33 e	1.01 ± 0.04 ef	9.90 ± 0.13 de
	*Rhizopus oligosporus* DSM 1964	48 h	8.14 ± 0.13 cd	55.22 ± 1.28 ef	57.43 ± 0.98 bcd	0.67 ± 0.02 bc	7.42 ± 0.09 b
		72 h	8.55 ± 0.07 d	60.65 ± 0.50 h	57.81 ± 0.71 bcd	0.94 ± 0.02 def	9.57 ± 0.15 d
		96 h	9.62 ± 0.10 ef	59.66 ± 0.85 gh	64.15 ± 1.61 e	0.91 ± 0.05 de	10.14 ± 0.15 de
	*Rhizopus oligosporus* NRRL 2710	48 h	8.25 ± 0.01 cd	56.81 ± 1.81 fg	60.84 ± 0.38 cde	0.78 ± 0.03 cd	8.45 ± 0.12 c
		72 h	8.24 ± 0.12 cd	52.74 ± 0.70 de	58.22 ± 1.14 bcd	1.13 ± 0.01 f	8.27 ± 0.15 c
		96 h	9.14 ± 0.11 e	59.44 ± 0.25 gh	61.53 ± 1.85 de	1.04 ± 0.07 ef	8.46 ± 0.22 c
	*Rhizopus oryzae* CBS 372.63	48 h	7.81 ± 0.11 c	50.40 ± 0.93 d	55.37 ± 0.84 bc	0.90 ± 0.02 de	8.46 ± 0.22 c
		72 h	8.06 ± 0.16 cd	43.05 ± 1.58 c	54.65 ± 1.03 b	1.05 ± 0.07 ef	8.79 ± 0.11 c
		96 h	7.96 ± 0.14 c	45.24 ± 0.19 c	56.72 ± 0.43 bcd	0.66 ± 0.05 bc	8.43 ± 0.22 c

One-way analysis of variance and Tuckey post-hoc test were applied. Data is shown as the mean ± SEM. Mean values within a column followed by different letters differ significantly (*p* ≤ 0.05). FCRS—Folin-Ciocalteu reacting substances; SA-ABTS˙^+^—ABTS˙^+^-scavenging assay; QA-ABTS˙^+^—Quencher-ABTS˙^+^ assay; SA-OH˙—OH˙-scavenging assay.

**Table 2:** Inositol phosphate profile (% relative peak area) of pumpkin oil-cake, autoclaved and fermented with *Rhizopus* strains.

			InsP1–2	InsP3	InsP4	InsP5	InsP6
Autoclaved oil-cake			1.32	4.74	6.11	18.98	68.84
Fermented oil-cake	Strain	time					
	*Rhizopus oligosporus* ATCC 64063	48 h	2.17	8.95	9.67	17.89	61.32
		96 h	0.83	6.02	11.27	23.56	58.34
	*Rhizopus oligosporus* DSM 1964	48 h	0.43	13.81	16.89	24.06	44.79
		96 h	1.04	5.45	7.1	19.61	66.78
	*Rhizopus oligosporus* NRRL 2710	48 h	0.98	27.25	8.8	6.24	56.72
		96 h	n.i.	4.23	2.15	14.87	78.75
	*Rhizopus oryzae* CBS 372.63	48 h	3.33	17.87	40.01	18.63	20.17
		96 h	1.97	12.92	37.08	21.54	26.47

InsP1–2—inositol mono and diphosphates, InsP3–InsP5—inositol tri-pentaphosphates, InsP6—inositol hexaphosphate; n.i.—not identified.
